# Clinical stage provides useful prognostic information even after pathological stage is known for prostate cancer in the PSA era

**DOI:** 10.1371/journal.pone.0234391

**Published:** 2020-06-11

**Authors:** Maxine M. Chen, Jaquelyn L. Jahn, John R. Barber, Misop Han, Meir J. Stampfer, Elizabeth A. Platz, Kathryn L. Penney

**Affiliations:** 1 Channing Division of Network Medicine, Brigham and Women’s Hospital and Harvard Medical School, Boston, MA, United States of America; 2 Department of Epidemiology, Harvard T.H. Chan School of Public Health, Boston, MA, United States of America; 3 Department of Social and Behavioral Sciences, Harvard T.H. Chan School of Public Health, Boston, MA, United States of America; 4 Department of Epidemiology, Johns Hopkins Bloomberg School of Public Health, Baltimore, MD, United States of America; 5 Department of Urology and the James Buchanan Brady Urological Institute, Johns Hopkins University School of Medicine, Baltimore, MD, United States of America; 6 Department of Nutrition, Harvard T.H. Chan School of Public Health, Boston, MA, United States of America; 7 Sidney Kimmel Comprehensive Cancer Center at Johns Hopkins, Baltimore, MD, United States of America; The Cancer Institute of New Jersey, Robert Wood Johnson Medical School, UNITED STATES

## Abstract

**Background:**

Pathological and clinical stage are associated with prostate cancer-specific survival after prostatectomy. With PSA screening, the post-surgery prognostic utility of clinical stage is debatable in studies seeking to identify new biomarkers. Few studies have investigated clinical stage and lethal prostate cancer association after accounting for pathological stage. We hypothesize that clinical stage provides prognostic information beyond pathological stage in the PSA era.

**Methods:**

Cox regression models tested associations between clinical and pathological stage and lethal prostate cancer among 3,064 participants from the Health Professionals Follow-Up Study and Physicians’ Health Study (HPFS/PHS) who underwent prostatectomy. Likelihood ratio tests and c-statistics were used to assess the models’ prognostic utility. Equivalent analyses were performed in 16,134 men who underwent prostatectomy at Johns Hopkins.

**Results:**

Independently, clinical and pathological stage were associated (p<0.0001 for both) with rate of lethal prostate cancer in HPFS/PHS. The model with clinical and pathological stage fit significantly better than the model with only pathological stage in all men (p = 0.01) and in men diagnosed during the PSA era (p = 0.04). The mutually adjusted model also improved discriminatory ability. In the Johns Hopkins cohort, the model with clinical and pathological stage improved discriminatory ability and fit significantly better overall (p<0.0001) and in the PSA era (p<0.0001).

**Conclusions:**

Despite stage migration resulting from widespread PSA screening, clinical stage remains associated with progression to lethal prostate cancer independent of pathological stage. Future studies evaluating associations between new factors and poor outcome following prostatectomy should consider including both clinical and pathological stages since the data is already available.

## Introduction

Although approximately 160,000 new cases of prostate cancer (PC) are estimated to occur in 2017, fewer than 30,000 are estimated to be lethal [1]. Identifying characteristics that distinguish potentially lethal PC (which we define as distant metastases or death from PC) from indolent PC is important in predicting prognosis at time of biopsy or primary treatment for subsequent surveillance and treatment decisions. Clinical stage, biopsy Gleason score, and serum prostate-specific antigen (PSA) levels, have long been used to predict pathological stage at diagnosis (pre-surgery) [2]. Studies have demonstrated the utility of Gleason score [3–5] and serum PSA [6,7] as pre-treatment prognostic markers. However, whether clinical stage provides useful information to predict lethal PC beyond pathological stage in men who have undergone prostatectomy for clinically localized disease is unclear.

The advent of PSA screening has led to migration towards lower clinical stage at diagnosis [8]. Due to the preponderance of lower risk tumors at diagnosis, there has been debate over the prognostic utility of clinical stage after pathological stage is known. With respect to post-surgery prognostic use in the PSA era, some have found that clinical staging was of no use in predicting the likelihood of adverse pathological outcomes (extraprostatic extension, positive surgical margins, and seminal vesicle invasion) [9], while others have observed that clinical stage improves prediction of progression and cancer-specific survival [10]. Clinical stage and pathological stage, while individually associated with cancer-specific survival after prostatectomy [11,12], are also correlated [13], which calls into question whether the inclusion of one with the other meaningfully improves prognostic utility. Few studies have published on the usefulness of clinical and pathological stages in predicting lethal PC survival after prostatectomy for clinically localized disease, and no study has evaluated whether clinical stage offers additional prognostic information beyond pathological stage in the same patients.

In the context of informing studies seeking to evaluate whether new exposures or markers at the time of prostatectomy are associated with subsequent poor outcome, we investigated the association of clinical stage and pathological stage with lethal PC survival after radical prostatectomy among 3,067 men from the Health Professionals Follow-up Study and the Physicians’ Health Study. We assessed whether clinical stage adds useful prognostic information when used with pathological stage and whether widespread PSA screening has diminished the prognostic utility of clinical stage for lethal PC progression in men surgically treated for clinically localized disease. Furthermore, we replicated these analyses in a clinical cohort of 16,134 men who underwent radical prostatectomy for localized PC at Johns Hopkins Hospital.

## Methods

### Study population

Model building was performed on a study population nested in the Health Professionals Follow-up Study (HPFS) and the Physicians’ Health Study (PHS). The model was replicated in a clinical cohort of men who underwent radical prostatectomy for clinically localized prostate cancer (PC) at Johns Hopkins (JH).

HPFS is an ongoing prospective cohort study of chronic diseases that enrolled 51,529 men from the United States (US) aged 40 to 75 years beginning in 1986 [14]. Men enrolled at baseline were free of diagnosed cancer, excluding non-melanoma skin cancer. Participants completed questionnaires regarding demographics, lifestyle, and medical history at baseline and biennially thereafter. PHS was a randomized, double-blind trial begun in 1982 to assess the effects of aspirin and beta-carotene on prevention of cardiovascular disease and cancer among 22,071 US male physicians aged 40 to 84 years at randomization (NCT00000500) [15]. Only men free of serious medical conditions were included in the trial. Participants completed questionnaires annually to ascertain information on diet, lifestyle behaviors, and medical history and biennially to ascertain health endpoints, including PC. Written informed consent was obtained for both studies. The Human Subjects Committees at Partners Healthcare and the Harvard T.H. Chan School of Public Health approved these studies.

The JH radical prostatectomy cohort included 23,721 men who underwent radical prostatectomy for clinically localized PC at Johns Hopkins between 1983 and 2014. Information on the men’s age, race, date of surgery, PSA at diagnosis, biopsy and pathological Gleason sums, and clinical and pathological stages were abstracted from medical records. We used data from the Johns Hopkins Master Prostatectomy Database, which stores clinical, pathological and demographic information under a consent waiver allowing its use for research without disclosing patient identifiers. The database is approved by the Johns Hopkins institutional review board, and meets the requirements of the Health Insurance Portability and Accountability Act. This analysis was approved by the IRB at the Johns Hopkins Bloomberg School of Public Health.

### Ascertainment of cases of prostate cancer and outcomes among the cases

In HPFS/PHS, study investigators confirmed self-reported PC diagnoses and extracted information on clinical stage, pathological stage, and clinical course by review of patient medical records and pathology reports. Many cases of PC in our cohorts predate currently used staging criteria, thus fine stage categories could not be consistently applied. For consistency across cohorts, we collapsed stage categories. For clinical stage, we used a three-category variable corresponding to clinically unapparent tumors (T1), prostate confined tumors (T2), and tumors that extend through the prostate and beyond (T3). For pathological stage, we used a four-category variable corresponding to tumor confined to organ (pT2), extra-prostatic extension or microscopic invasion of bladder neck (pT3a), seminal vesicle invasion (pT3b), and metastases to regional lymph nodes (pN1). Medical records obtained during follow-up were the primary source of Gleason scores. For 33.3% of patients, Gleason scores were obtained from formalin-fixed paraffin-embedded radical prostatectomy (RP) specimens re-graded by a single pathologist using the 2005 International Society of Urological Pathology revised criteria.

The outcome of lethal PC encompasses deaths from PC and progression to bony or other distant metastases. Deaths are ascertained through repeated mailings, telephone calls, and search of the National Death Index. Cause of death is assigned after review of medical records, death certificates (underlying cause), and family information. Evidence of distant metastases was required for establishing PC death. Follow-up for mortality was >99% complete in the PHS and 98% complete in the HPFS. Metastases development is determined from self-reported follow-up questionnaires and review through contact with the patients’ treating physicians or medical records.

In the JH radical prostatectomy clinical cohort, all men had a biopsy-confirmed PC diagnosis, and were surgically treated at JH. The attending surgeon assigned clinical stage using The American Joint Committee on Cancer staging guidelines [16]. For clinical grade, the Gleason sum for the biopsy core with the highest grade was used for analysis. Resected prostate and seminal vesicles were pathologically evaluated. Pelvic lymph node dissection was usually performed unless the chance of lymph node involvement is low; all pelvic lymph nodes were sectioned for cancer. Pathological stage was classified as follows: confined to the prostate (organ confined/pT2); cancer outside of the prostate but without seminal vesicle or lymph node involvement (extraprostatic extension/pT3a); cancer invaded the seminal vesicles but not the lymph nodes (seminal vesicle involvement/pT3b); and cancer in the dissected pelvic lymph nodes (lymph node involvement/pN1). The two primary Gleason patterns in the dominant tumor focus was determined using the 2005 International Society of Urological Pathology modified grading system [17]. The stages were then grouped as for the HPFS/PHS cohorts.

Men were clinically followed to 2014. Metastasis was identified by bone scan, CT scan or MRI. Date and cause of death were obtained from the US Social Security Administration’s Death Master File and/or from the National Death Index. Men with PC recorded as the underlying cause of death were considered to have died of PC. We excluded men with missing clinical or pathological stage, men lost to follow-up, and men diagnosed in 2014 because of short follow-up time. Men with clinical M1 stage disease were ineligible for this analysis because they are not typically candidates for radical prostatectomy. After exclusions, the analytic cohort included 16,134 men among whom 811 developed lethal disease defined as the development of distant metastases (bone or soft tissue) or death from PC as the underlying cause. Because of lack of proportional hazards, we truncated follow-up time at ≤20 years, which left 772 lethal cases for this analysis.

### Statistical analysis

Statistical analyses in the HPFS/PHS cohorts included only men treated with prostatectomy, without known metastases at time of diagnosis, and with both clinical and pathological stage information (n = 3,064). We used Cox proportional hazards regression models to calculate hazard ratios (HRs) and 95% confidence intervals (CIs) for the association of lethal PC with clinical stage, pathological stage, and both clinical and pathological stage. We performed secondary analyses stratified by pre- and post-PSA era (after 1990) and adjusted all models for age at diagnosis and year of prostatectomy. To evaluate model fit, we performed likelihood ratio tests (LRT). We measured each model’s discriminatory power with the c-statistic. [18] The same models were employed in the JH radical prostatectomy cohort.

All analyses were performed using SAS version 9.4 (SAS Institute Inc., Cary, NC, US). Statistical significance determined from two-sided tests was set at p<0.05.

## Results

### HPFS and PHS cohorts

Clinical characteristics of prostate cancer (PC) cases included in this study from HPFS/PHS are described in **Table 1**. We followed 3,064 men with clinical and pathological stage for a median of 13.7 years. Of these men, 222 had lethal PC and were followed for a median of 11.4 years. Median age at PC diagnosis was 66.0 years among all cases and 65.9 years among those who had lethal PC. The majority of men were diagnosed in the prostate-specific antigen (PSA) screening era (93.1% of all cases, 76.6% of lethal PC cases). Men with lethal PC had higher median PSA at diagnosis than men without lethal PC (8.6 and 6.1 ng/mL, respectively) and higher Gleason scores at diagnosis. Clinical stage and pathological stage were significantly, though weakly, correlated (Pearson r = 0.22, p <0.0001, **S1 Table**).

**Table 1:** Clinical characteristics of men diagnosed with prostate cancer who underwent radical prostatectomy for clinically localized prostate cancer in HPFS, PHS, and the JH RRP cohort.

	HPFS and PHS^1^		JH RRP cohort^2^	
	All Cases^3^ n = 3064	Lethal PC^4^ n = 222	All Cases^3^ n = 16,134	Lethal PC^4^ n = 772
Median age at time of diagnosis	66.0	65.9	59	60
IQR	62.0, 69.8	61.0, 69.4	54.0, 63.0	55.0, 64.0
Median follow-up time, years^5^	13.75	11.1	5.0	7.0
IQR	9.5, 18.3	6.7. 15.8	2.0, 11.0	3.0, 11.0
Gleason score at diagnosis (%)				
≤6	1879 (61.3)	71 (32.0)	11261 (69.8)	255 (33.0)
7	792 (25.9)	79 (35.6)	3,971 (24.6)	322 (41.7)
8	151 (4.9)	29 (13.1)	560 (3.5)	106 (13.8)
9	67 (2.2)	15 (6.7)	258 (1.6)	68 (8.8)
10	9 (0.3)	3 (1.3)	16 (0.1)	4 (0.5)
NA	166 (5.4)	25 (11.3)	68 (0.4)	17 (2.2)
Median PSA at diagnosis (ng/mL)	6.1	8.6	4.9	6.6
IQR	4.5, 9.1	5.3, 15.0	3.2, 7.3	3.4, 11.4
PSA era				
Diagnosed before 1990 (%)	135 (4.4)	40 (18.0)	965 (6.0)	181 (23.5)
Diagnosed in 1990 or later (%)	2929 (95.6)	182 (82.0)	15,169 (94.0)	591 (76.5)
Clinical stage				
cT1	1808 (59.0)	83 (37.4)	10760 (66.7)	212 (27.5)
cT2	1182 (38.6)	113 (50.9)	5234 (32.4)	509 (65.9)
cT3+	74 (2.4)	26 (11.7)	140 (0.9)	51 (6.6)
Pathological stage				
pT2	2299 (75.0)	91 (41.0)	10,486 (65.0)	95 (12.3)
pT3a	499 (16.3)	49 (22.1)	4,446 (27.6)	295 (38.2)
pT3b	202 (6.6)	56 (25.2)	721 (4.5)	189 (24.5)
pT4/pN1	64 (2.1)	26 (11.7)	481 (3.0)	193(25.0)
Number of all-cause deaths	772 (25.2)	---	2215 (13.7)	---
Number of PC deaths	129 (9.3)	129 (72.5)	503 (3.1)	503 (65.2)

1) Restricted to men with a clinical and pathologic stage available. Excluded men with clinical stage M1 because they are not typically treated by radical prostatectomy.

2) Restricted to men with a clinical and pathologic stage available, not lost to follow-up, and had surgery before 2014. Excluded men with clinical stage M1 because they are not typically treated by radical prostatectomy.

3) Includes men with lethal PC.

4) Men with confirmed PC as cause of death or the development of distant metastases to bone or soft tissue.

5) Follow-up was truncated at ≥ 20 years for JH RRP cohort.

To identify the best fitting model for lethal PC, we performed time-to-event analyses of clinical stage alone, pathological stage alone, and clinical and pathological stage together (**Table 2)**. Clinical stage was significantly associated (p<0.0001) with rate of lethal PC. Men with T2 clinical stage had 1.51 times the rate of lethal PC compared to men with T1 clinical stage (95% CI: 1.12, 2.04) and men with T3 clinical stage had 4.25 times the rate of lethal PC (95% CI: 2.65, 6.82). Pathological stage was also significantly associated (p<0.0001) with rate of lethal PC. Compared to men with T2 pathological stage, men with T3a, T3b, and N1 pathological stage had 2.28 (95% CI: 1.61, 3.23), 7.59 (95% CI: 5.41, 10.62), and 9.04 (95% CI: 5.81, 14.05) times the rate of lethal PC, respectively. To determine whether clinical stage added useful information after accounting for pathological stage, we included both in a mutually adjusted time-to-event analysis. In this model, both stage classifications were independently associated with lethal PC. In a likelihood ratio test, we found a significantly better fit (p = 0.01) for the model including clinical and pathological stage compared to the model with pathological stage alone. Furthermore, the discriminative ability of the mutually adjusted model (c-statistic = 0.73) was greater than that for clinical stage alone (c-statistic = 0.55) and pathological stage alone (c-statistic = 0.72).

**Table 2:** Association of clinical and pathologic staging with risk of lethal prostate cancer among men who underwent radical prostatectomy for clinically localized prostate cancer, HPFS and PHS, 1981–2015.

	N lethal cases/N non-lethal cases	HR		95% CI	- 2 Log L	C-Statistic
Model*						
Clinical stage						
cT1	83/1725	Ref.		-	3209.8	0.55
cT2	113/1069	1.51		(1.12–2.04)		
cT3	26/48	4.25		(2.65–6.82)		
Pathological stage						
pT2	91/2208	Ref.		-	3084.7	0.72
pT3a	49/450	2.28		(1.61–3.23)		
pT3b	56/146	7.59		(5.41–10.62)		
pN1	26/38	9.04		(5.81–14.05)		
Clinical and pathological stages						
cT1	83/1725	Ref.		-	3076.2	0.73
cT2	113/1069	1.45		(1.08–1.96)		
cT3	26/48	1.88		(1.13–3.12)		
pT2	91/2208	Ref.		-		
pT3a	49/450	2.12		(1.48–3.03)		
pT3b	56/146	7.44		(5.31–10.44)		
pN1	26/38	7.48		(4.61–12.12)		
Likelihood Ratio Tests			Difference from Full Model			P-value
Clinical stage added to pathologic stage			8.4			0.0148
Pathologic stage added to clinical stage			133.6			<0.0001

*Models adjusted for age and year of RRP

We conducted time-to-event analyses stratified by PSA era to evaluate whether stage migration due to PSA screening influences the utility of clinical stage **(Table 3)**. Clinical stage was not significantly associated (p = 0.97) with rate of lethal PC among men diagnosed with PC before 1991 while pathological stage was significantly associated (p = 0.0002). Clinical stage did not significantly improve model fit when adjusted with pathological stage compared to pathological stage alone (LRT p = 0.99) among men diagnosed before the PSA era. Similarly, there was no meaningful difference in discriminative ability between the model with only pathological stage and the mutually adjusted model. However, among men diagnosed with PC during the PSA era, both clinical stage alone and pathological stage alone were significantly associated with rate of lethal PC (both p<0.0001). Adding clinical stage to the model along with pathological stage significantly improved model fit (LRT p = 0.03) compared to pathological stage alone among cases diagnosed during the PSA era. The discriminative ability of the mutually adjusted model (c-statistic = 0.74) was greater than the discriminative abilities of the pathological stage only model (c-statistic = 0.73) and the clinical stage only model (c-statistic = 0.61).

**Table 3:** Association of clinical and pathologic staging with risk of lethal prostate cancer among men who underwent radical prostatectomy for clinically localized prostate cancer, stratified by PSA era, HPFS and PHS, 1981–2015.

	Pre-PSA era (1981–1990)					PSA era (1990–2015)				
	N lethal cases/N non-lethal cases	HR	95% CI	- 2 Log L	C-Statistic	N lethal cases/N non-lethal cases	HR	95% CI	- 2 Log L	C-Statistic
Model*										
Clinical stage										
cT1	6/16	Ref.	-	349.4	0.51	77/1709	Ref.	-	2651.2	0.61
cT2	28/69	1.02	(0.42–2.47)			85/1000	1.51	(1.10–2.08)		
cT3	6/10	1.13	(0.36–3.51)			20/38	5.83	(3.48–9.76)		
Pathological stage										
pT2	19/59	Ref.	-	333.4	0.60	72/2149	Ref.	-	2536.3	0.73
pT3a	6/23	1.01	(0.40–2.55)			43/427	2.69	(1.84–3.93)		
pT3b	14/9	4.49	(2.21–9.09)			42/137	8.03	(5.48–11.78)		
pN1	1/4	1.13	(0.15–8.59)			25/34	12.77	(8.04–20.27)		
Clinical & pathological stages										
cT1	6/16	Ref.	-	333.4	0.60	77/1709	Ref.	-	2529.1	0.74
cT2	28/69	1.05	(0.43–2.55)			85/1000	1.40	(1.02–1.93)		
cT3	6/10	1.11	(0.32–3.85)			20/38	2.00	(1.14–3.53)		
pT2	19/59	Ref.	-			72/2149	Ref.	-		
pT3a	6/23	0.99	(0.34–2.84)			43/427	2.49	(1.69–3.67)		
pT3b	14/9	4.45	(2.16–9.17)			42/137	7.87	(5.36–11.55)		
pN1	1/4	1.09	(0.13–9.04)			25/34	10.08	(6.01–16.92)		
Likelihood Ratio Tests		Difference from Full Model			P-value		Difference from Full Model			P-value
Clinical stage added to pathologic stage		0			0.9866		7.2			0.0268
Pathologic stage added to clinical stage		16			0.0012		122.1			<0.0001

*Models adjusted for age and year of RRP

### Johns Hopkins radical prostatectomy cohort

These models were replicated in an independent cohort of 16,134 men who underwent radical prostatectomy for clinically localized PC at Johns Hopkins **(clinical characteristics described in Table 1)** to verify that clinical stage improved model fit when added to pathological stage. Of these men, 772 developed lethal PC. The median follow-up was 5 years for all cases and 7 years for men who developed lethal PC. Median age at the time of radical prostatectomy was 59 years for all cases and 60 years for cases with lethal PC. Correlation between clinical and pathological stage was also significant, though weak, in this cohort **(**Pearson r = 0.27, p <0.0001, **S2 Table)**. The model including both clinical stage and pathological stage performed significantly better (LRT p<0.0001) than the model with only pathological stage **(Table 4)**. Additionally, the discriminatory power of the mutually adjusted model (c-statistic = 0.83) was greater than that of the models with clinical stage alone (c-statistic = 0.68) and pathological stage alone (c-statistic = 0.82). Even when stratified by pre- and post-PSA era, the model with both clinical and pathological stage fit significantly better (pre-PSA p = 0.03, post-PSA p<0.0001) than the model with only pathological stage **(Table 5)**. Though the discriminatory power of the model with both clinical and pathological stage remained the same as the model with pathological stage alone in the pre-PSA era, the discriminatory power of the mutually adjusted model (c-statistic = 0.84) was greater than that of the models with pathologic stage alone (c-statistic = 0.80) and clinical stage alone (c-statistic = 0.67) in the PSA era.

**Table 4:** Association of clinical and pathologic staging with risk of lethal prostate cancer among men who underwent radical prostatectomy for clinically localized prostate cancer, JH RRP Cohort, 1983–2014*.

	N lethal cases/N non-lethal cases	HR		95% CI	- 2 Log L	C-Statistic
Model**						
Clinical stage						
cT1	212/10,548	Ref.		-	8734.6	0.68
cT2	509/4,725	3.40		(2.86–4.04)		
cT3	51/89	11.35		(8.24–15.64)		
Pathological stage						
pT2	95/10,391	Ref.		-	7837.7	0.82
pT3a	295/4,151	5.49		(4.34–6.95)		
pT3b	189/532	27.48		(21.38–35.33)		
pN1	193/288	40.49		(31.44–52.16)		
Clinical and pathological stages						
cT1	212/10,548	Ref.		-	7750.8	0.83
cT2	509/4,725	2.06		(1.73–2.46)		
cT3	51/89	3.53		(2.54–4.92)		
pT2	95/10,391	Ref.		-		
pT3a	295/4,151	4.70		(3.71–5.97)		
pT3b	189/532	22.14		(17.14–28.59)		
pN1	193/288	31.87		(24.61–41.26)		
Likelihood Ratio Tests			Difference from Full Model			P-value
Clinical stage added to pathologic stage			87			<0.0001
Pathologic stage added to clinical stage			983.8			<0.0001

*Follow-up was truncated at > 20 years.

**Models adjusted for age and year of RRP.

**Table 5:** Association of clinical and pathologic staging with risk of lethal prostate cancer among men who underwent radical prostatectomy for clinically localized prostate cancer, stratified by PSA era, JH RRP Cohort, 1983–2014*.

	Pre-PSA era (1981–1990)					PSA era (1990–2015)				
	N lethal cases/N non-lethal cases	HR	95% CI	- 2 Log L	C-Statistic	N lethal cases/N non-lethal cases	HR	95% CI	- 2 Log L	C-Statistic
Model**										
Clinical stage										
cT1	13/141	Ref.	-	1821.5	0.61	199/10,407	Ref.	-	6808.1	0.67
cT2	158/627	2.4	(1.38–4.32)			351/4,098	3.49	(2.92–4.18)		
cT3	10/16	6.2	(2.66–14.29)			41/73	14.04	(9.85–20.02)		
Pathological stage										
pT2	20/317	Ref.	-	1622.9	0.77	75/10,074	Ref.	-	6092.0	0.80
pT3a	61/405	2.3	(1.37–3.77)			234/3,746	6.73	(5.17–8.75)		
pT3b	41/36	12.2	(7.12–21.06)			148/496	32.32	(24.38–42.85)		
pN1	59/26	22.9	(13.62–38.66)			134/262	46.64	(34.91–62.31)		
Clinical & pathological stages										
cT1	13/141	Ref.	-	1618.4	0.77	199/10,407	Ref.	-	6003.4	0.84
cT2	158/627	1.7	(0.94–3.05)			351/4,098	2.09	(1.74–2.52)		
cT3	10/16	2.3	(0.98–5.59)			41/73	4.28	(2.97–6.16)		
pT2	20/317	Ref.	-			75/10,074	Ref.	-		
pT3a	61/405	2.0	(1.21–3.39)			234/3,746	5.77	(4.42–7.52)		
pT3b	41/36	10.8	(6.23–18.79)			148/496	25.52	(19.15–34.01)		
pN1	59/26	20.7	(12.18–35.20)			134/262	35.55	(26.45–47.78)		
Likelihood Ratio Tests		Difference from Full Model			P-value		Difference from Full Model			P-value
Clinical stage added to pathologic stage		4.5			0.03		88.6			<0.0001
Pathologic stage added to clinical stage		203.1			<0.0001		804.8			<0.0001

*Follow-up was truncated at > 20 years.

**Models adjusted for age and year of RRP.

## Discussion

Both clinical stage and pathological stage were significantly associated with the development of lethal prostate cancer (PC) among men with stage information who underwent radical prostatectomy for clinically localized disease. Though pathological stage was much more strongly associated with lethal PC than clinical stage, clinical stage still provided statistically significant prognostic information beyond pathological stage for lethal PC in our cohorts, improving model fit and discriminatory power. Clinical stage also remained useful after widespread PSA screening, providing statistically significant additional information to pathological stage in men diagnosed with PC after 1990.

Studies have provided conflicting evidence regarding the utility of clinical stage for prognosis, alternately concluding that clinical stage fails to predict pathological stage consistently [19], that it can be a surrogate for post-surgical pathological stage and risk of recurrence [20,21], and that it fails to predict post-surgical biochemical recurrence [9]. Although we did find that clinical stage and pathological stage were statistically significantly correlated, the strength of that correlation was low. Our study provides evidence that clinical stage is still of use in modeling the rate of lethal PC after surgical treatment, improving both discrimination and model performance when included with pathological stage in most models tested. Our study differs from previous studies due to our lengthy follow-up (median 13.7 years among all cases in the HPFS/PHS cohorts and 5 years in the JH cohort) and the use of lethal PC as our outcome. Biochemical recurrence is often an imperfect identifier of the lethal potential of PC given that most men with biochemical recurrence do not die of PC [22]. Thus, lethal PC is a more clinically relevant measure of progression.

Since PSA screening began in the US in the early 1990s, there has been earlier detection of PC leading to migration to lower clinical stages at time of diagnosis, even in high-risk patients [23]. This has called into question whether clinical stage meaningfully contributes to identifying those at higher risk of poor outcome after pathological stage is known. However, we found that clinical stage (both before and after taking into account pathological stage) was more strongly associated with lethal PC in the PSA era than before widespread PSA screening in HPFS/PHS. Our results were recapitulated in the larger Johns Hopkins prostatectomy cohort, which included cases back to 1983. Similarly, we saw that clinical stage improved model performance and discrimination when added to pathological stage in the PSA era in both the HPFS/PHS cohorts and the JH cohort. A potential explanation for the additional prognostic value of clinical stage in the PSA era is that higher clinical stage at diagnosis indicates that the PC was unlikely diagnosed from PSA detection and more likely to be further along in disease progression. Those diagnosed via PSA screening are likely to have better outcomes. Thus, clinical stage greater than T1 may point to a higher risk of unfavorable outcomes than if one were PSA diagnosed. The additional prognostic information provided by clinical stage confirms its value as a resource when optimizing models for post-surgery PC prognosis. Though the improvement in prognostication is small, there is no cost to including clinical stage data because it is already available. Investigators seeking new prognostic tools for PC should consider whether other markers only provide the same information as data already available.

Our study was limited by variations in stage assessment, which was performed by several providers, within and between the cohorts and over time. Previous studies have reported low inter-observer consistency in both clinical [24] and pathologic [25] staging of PC. Furthermore, stage sub-categories used in the HPFS, PHS and JH cohorts were not consistent. To offset this issue and make categories between the two studies comparable, we collapsed sub-categories of stage into the main stage categories. Thus, we were unable to evaluate sub-categories of stage for a more detailed analysis. Since the cohorts used in these analyses primarily consist of white men, our study may be of limited in generalizability to other patient groups. Studies in other populations, such as in men of African descent, should be conducted in order to assess the importance of clinical stage beyond pathological stage in patient groups with higher likelihood of progression to lethal disease. We additionally have no information on the use of imaging to guide biopsies and define clinical stage; with the years of diagnosis in these studies, few would have had an MRI-guided biopsy. A future study should explore the performance of clinical stage assigned by MRI with pathological stage in a prognostic model.

## Conclusions

Clinical stage, when used together with pathological stage, significantly improves both discrimination and model performance over pathological stage alone for lethal PC even after the advent widespread PSA screening, which has led to migration to lower clinical stages at diagnosis. Our findings from epidemiologic and clinical cohorts highlight that clinical stage provides useful prognostic information beyond pathological stage about lethal disease risk after prostatectomy for clinically localized disease. Though the contribution of clinical stage is relatively small, the data is often already collected, of no additional cost, and readily available for inclusion. Studies evaluating whether new exposures or markers at the time of surgery provide prognostic information about later risk of poor outcome following prostatectomy should consider including both clinical and pathological stages to capture the already available prognostic information.

## Supporting information

S1 Table Clinical stage by pathologic stage, categorical variables in HPFS and PHS. (DOCX)

S2 Table Clinical stage by pathologic stage, categorical variables in JH RRP cohort. (DOCX)
