# Supplementary material for: Clinical stage provides useful prognostic information even after pathological stage is known for prostate cancer in the PSA era
Source: PLoS One. 2020 Jun 11;15(6):e0234391. doi: 10.1371/journal.pone.0234391 (PMC7289430; doi:10.1371/journal.pone.0234391)
Supplement: S1 Table — (DOCX) [file pone.0234391.s001.docx]

**S1 Table. Clinical stage by pathologic stage, categorical variables in HPFS and PHS**

| **All Cases** |  | Pathologic Stage | | | | |
| --- | --- | --- | --- | --- | --- | --- |
| Clinical Stage |  | PT2 | PT3a | PT3b | PN1 | total |
|  | CT1 | 1445 (47.2) | 245 (8.0) | 101 (3.3) | 17 (0.6) | 1808 (59.0) |
|  | CT2 | 850 (27.7) | 220 (7.2) | 87 (2.9) | 25 (0.8) | 1182 (38.6) |
|  | CT3+ | 4 (0.1) | 34 (1.1) | 14 (0.5) | 22 (0.7) | 74 (2.4) |
|  | Total | 2299 (75.0) | 499 (16.3) | 202 (6.6) | 64 (2.1) | 3064 (100) |
| Pearson correlation between clinical and pathologic stages: | | | | | 0.22 (p<0.0001) | |
