# Supplementary material for: Clinical stage provides useful prognostic information even after pathological stage is known for prostate cancer in the PSA era
Source: PLoS One. 2020 Jun 11;15(6):e0234391. doi: 10.1371/journal.pone.0234391 (PMC7289430; doi:10.1371/journal.pone.0234391)
Supplement: S2 Table — (DOCX) [file pone.0234391.s002.docx]

**S2 Table. Clinical stage by pathologic stage, categorical variables in JH RRP Cohort**

| **All Cases** |  | Pathologic Stage | | | | |
| --- | --- | --- | --- | --- | --- | --- |
| Clinical Stage |  | PT2 | PT3a | PT3b | PN1 | total |
|  | CT1 | 11516 (51.6) | 3196 (14.3) | 433 (1.9) | 186 (0.8) | 15331 (68.69) |
|  | CT2 | 3414 (15.3) | 2631 (11.8) | 442 (2.0) | 338 (1.5) | 6825 (30.6) |
|  | CT3+ | 22 (0.1) | 80 (0.4) | 27 (0.1) | 33 (0.2) | 162 (0.7) |
|  | Total | 14952 (67.0) | 5907 (26.4) | 902 (4.0) | 557 (2.5) | 22318 |
| Pearson correlation between clinical and pathologic stages: | | | | | 0.27 (p<0.0001) | |
